# Comparative Study of SVM Methods Combined with Voxel Selection for Object Category Classification on fMRI Data

**DOI:** 10.1371/journal.pone.0017191

**Published:** 2011-02-16

**Authors:** Sutao Song, Zhichao Zhan, Zhiying Long, Jiacai Zhang, Li Yao

**Affiliations:** 1 State Key Laboratory of Cognitive Neuroscience and Learning, Beijing Normal University, Beijing, China; 2 School of Information Science and Technology, Beijing Normal University, Beijing, China; Cuban Neuroscience Center, Cuba

## Abstract

**Background:**

Support vector machine (SVM) has been widely used as accurate and reliable method to decipher brain patterns from functional MRI (fMRI) data. Previous studies have not found a clear benefit for non-linear (polynomial kernel) SVM versus linear one. Here, a more effective non-linear SVM using radial basis function (RBF) kernel is compared with linear SVM. Different from traditional studies which focused either merely on the evaluation of different types of SVM or the voxel selection methods, we aimed to investigate the overall performance of linear and RBF SVM for fMRI classification together with voxel selection schemes on classification accuracy and time-consuming.

**Methodology/Principal Findings:**

Six different voxel selection methods were employed to decide which voxels of fMRI data would be included in SVM classifiers with linear and RBF kernels in classifying 4-category objects. Then the overall performances of voxel selection and classification methods were compared. Results showed that: (1) Voxel selection had an important impact on the classification accuracy of the classifiers: in a relative low dimensional feature space, RBF SVM outperformed linear SVM significantly; in a relative high dimensional space, linear SVM performed better than its counterpart; (2) Considering the classification accuracy and time-consuming holistically, linear SVM with relative more voxels as features and RBF SVM with small set of voxels (after PCA) could achieve the better accuracy and cost shorter time.

**Conclusions/Significance:**

The present work provides the first empirical result of linear and RBF SVM in classification of fMRI data, combined with voxel selection methods. Based on the findings, if only classification accuracy was concerned, RBF SVM with appropriate small voxels and linear SVM with relative more voxels were two suggested solutions; if users concerned more about the computational time, RBF SVM with relative small set of voxels when part of the principal components were kept as features was a better choice.

## Introduction

It has long been a great interest of human being to make tremendous efforts to explore the mysterious working of the human brain, especially its possible coding schemes and interactions with the real world. With the most recently advanced neuroimaging techniques such as electroencephalogram (EEG) and functional magnetic resonance imaging (fMRI), the somewhat superstitious mind-reading is starting to convert to a real science. EEG records the electrical potential by attaching a number of electrodes to a person's scalp. Numerous studies have demonstrated correlations between EEG signals and mental tasks, such as active counting, active attention work or movement imagination [1]–[4]. Although advances in electrophysiological recording methods nowadays employ intrusive technologies, providing EEG with high topographical resolution, EEG has a poor spatial (centimeter) resolution which makes it inappropriate for the study of high-level cognitive activities involved with multiple cortices. fMRI offers further option to look into the brain function over its whole volume with reasonable spatial resolution (millimeter), and to research the relationship between the sensory world and the representation of complex objects in the brain. Using the approaches reviewed in Norman et al. [5], the fMRI data acquired were used to decode the neural representation of different categories of objects [6], [7], to discriminate the orientation of a striped pattern being viewed by a study participant [8], [9], or to predict human brain activity associated with the meanings of nouns [10].

The two basic and important procedural steps in analyzing fMRI data to distinguish cognitive states are feature selection (voxel selection) and feature based classification. Voxel selection is widely used for efficient classifications and an issue to be discussed in great depth in this study. Because of the information redundancy, only a subset of the brain voxels determined by voxel-wise univariate approaches or few characteristic patterns identified by multivariate techniques are needed [11]–[13]. For the univariate approaches, there are some voxel selection methods available to reduce the dimension [14]–[16]. For example, a common approach was to choose voxels in the whole brain or regions of interest (ROI) based on the discrimination ability or activity of the voxels, and the number of voxels used for discrimination has to be decided according to the discrimination ability or the active intensity before classification. Apart from the way of minimizing the average error rate across subjects [15], multiple comparison criterions [14], [16], such as false discovery rate (FDR) or family wise error (FWE) correction, were used to set the number of the voxels. In this way, all the significantly active voxels can all be used for classification. However, in some conditions, the multiple comparison correction method (e.g. FWE correction) may be too strict and no significantly active voxels at a given level can be found. To address this issue, voxels can be selected with a threshold not corrected for multiple comparisons. The reliability of this kind of voxel selection method, however, has yet to be addressed. Until now, no study has given comparative empirical results on selecting active voxels with or without multiple comparison correction through the whole brain or ROIs.

The subsequent feature classification acts as the “decoding function”, which convert the feature vector assembled from selected voxel set into a meaningful brain state. Different from the traditional univariate analysis methods which treated each voxel as a separate entity and were statistically inference oriented, the multivariate statistical machine learning algorithms most commonly used in the mind-reading community were designed to learn the statistical regularities of the data set, and then performed the prediction or classification of brain states from observed fMRI data based on the regularities [5], [6], [10], [14]–[18]. For example, Cox and Savoy [14] used linear discriminant analysis (LDA), linear support vector machines (SVM) and cubic polynomial kernel SVM to classify patterns of fMRI activation evoked by the visual presentation of 10 categories of objects, and the average of scans in a block (20s worth) was treated as a single example to perform the discrimination. Carlson et al. [6] applied fisher linear discriminant (FLD) method to discriminate patterns of activity in the categorical representation of 3 objects (houses, faces and chairs) of single scan. Mourão-Miranda et al. [17] compared the performance of FLD and linear SVM in the classification of two attention-required tasks: face matching and location matching with the single scan data acquired over 3.6s. Among those machine learning methods, SVM was demonstrated to be most effective, and the classifier can predict the brain states using data of a single block lasted 20s, or even a single scan, with TRs of only several seconds. Although non-linear SVM extends the linear SVM by constructing a rich set of non-linear decision functions and many conceivable sources of nonlinearity in neural signals exist, the non-linear SVM did not outperform linear one as demonstrated in previous study on fMRI data [14]. The researcher pointed out two possible reasons: (1) the fundamental linear separability characteristic existed in distributed patterns itself of fMRI activity evoked by the visual presentation of various categories of objects, or (2)the non-linear cubic polynomial kernel used for classification did not capture the non-linear character of the data. The second reason speaks for the need of an effective classification model for neuroimaging data analysis. More recent studies give implicit comparative study about the performances of multivariate classifiers in decoding the category of visual objects from fMRI data [18], in producing or evaluating information maps [12], [13], including two kernels SVM-linear and radial basis function kernel SVM (RBF SVM), which are what we are interested in the present study.

Although classification accuracy and the amounts of time needed for classification have been extensively used in previous studies to evaluate the performance of fMRI analysis methods, it is rarely addressed the problem of how to design classifier combined with voxel selection to reach the optimal overall performance. Thus, this study focuses on the examination of the computational effectiveness and computation time of linear and non-linear SVM for fMRI classification together with voxel selection schemes. Data used in this study are from a visual stimulus representation experiment for which subjects did simple one-back repetition detection task when objects from 4 categories (faces, houses, cars and cats) were presented. We compared linear and RBF SVM under each of six different voxel selection methods (see *Voxel selection schemes* under *Materials and methods*). In addition, as a commonly used approach to reduce the dimensions of the feature space, the effect of principal component analysis (PCA) was also investigated in classification accuracy and computation time. Our results demonstrated that, (1) Voxel selection had an important impact on the performance of the classifiers: in a relative low dimensional feature space, RBF SVM outperformed linear SVM significantly; in a relative high dimensional space, linear SVM performed better than its counterpart; (2) Considering the classification accuracy and the amounts of time needed together, when all the selected voxels were treated as features, an effective classification result could be achieved by linear SVM with large number of voxels; when part of the principal components (PCs) of the input voxel space were kept as features, the computational efficiency was improved, and an effective classification result could be achieved by non-linear RBF SVM with a small set of voxels. These results may be informative to researchers to choose classifiers with a specific voxel selection method to achieve the desired accuracy or efficiency.

## Results

**Figure 1:**
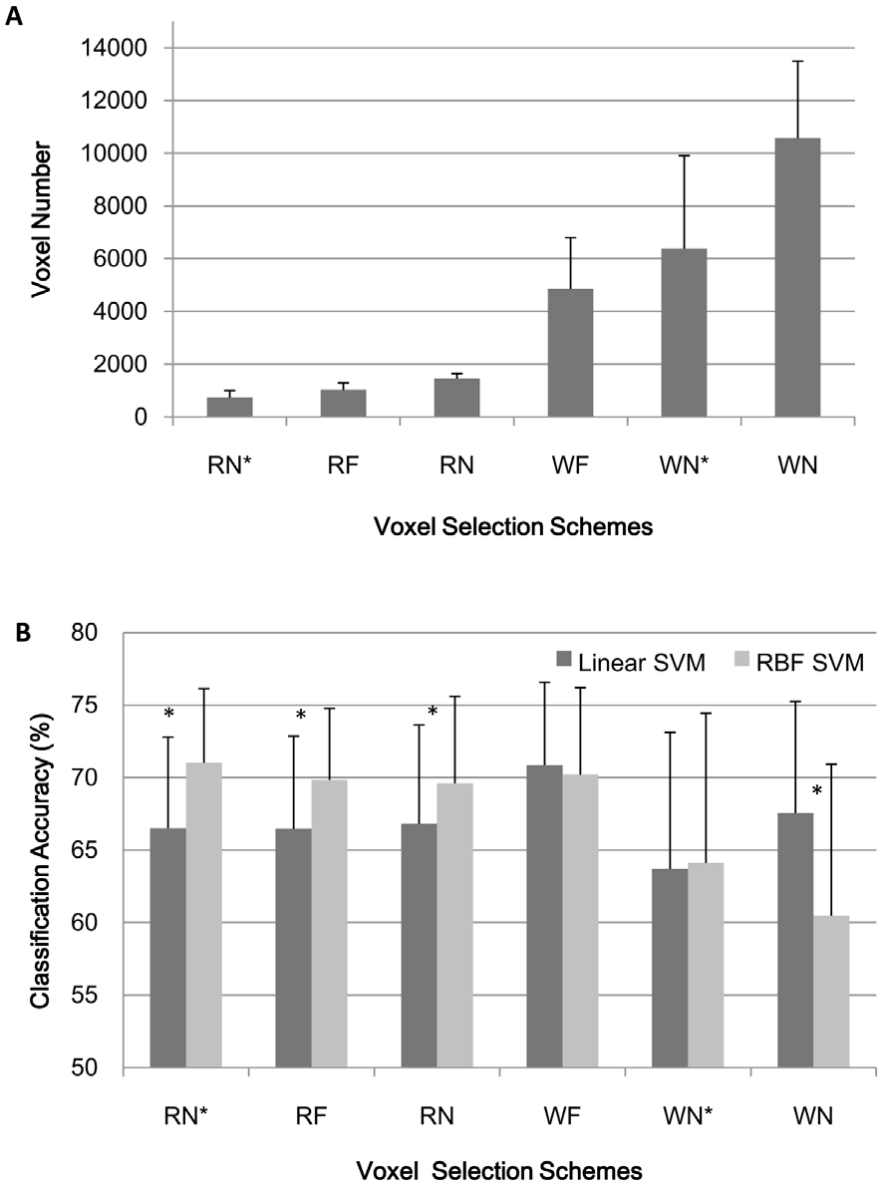
The number of voxels and classification accuracy of linear and RBF SVM without PCA. (A) The number of voxels under different voxel selection methods. (B) The mean classification accuracy across 14 subjects of linear and non-linear (RBF) SVM under each of the six different voxel selection methods when the features were the selected voxels: The Friedman test indicated significant difference among the six voxel selection methods for linear SVM and RBF SVM. The Wilcoxon signed-rank test indicated RBF SVM outperformed linear SVM under RN*, RF and RN methods; while linear SVM outperformed its counterpart under WN method; no significant difference existed between linear and RBF SVM under WF and WN* voxel selection methods. Description of the six voxel selection methods can be found in the text. * represent significant difference exists between linear and RBF SVM classification results.

**Figure 2:**
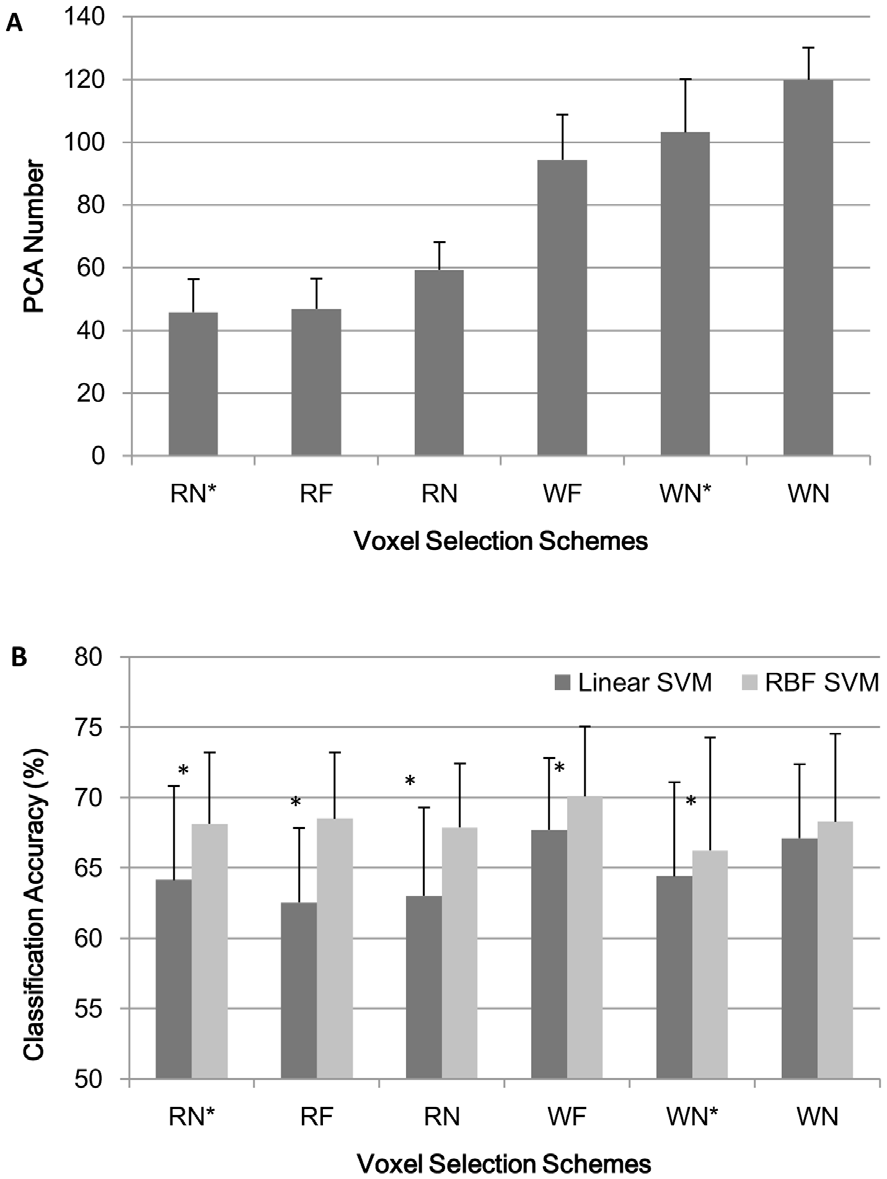
The number of PCs and classification accuracy of linear and RBF SVM with PCA. (A) The number of PCs when 95% variance of the original data was kept for the subsequent classification. (B) The mean classification accuracy across 14 subjects of linear and non-linear (RBF) SVM under each of the six different voxel selection methods when the features were PCs determined by the PCA procedure: The Friedman test indicated significant difference among the six voxel selection methods for linear SVM and non-significant difference for RBF SVM. RBF SVM outperformed linear SVM under all the voxel selection methods. Description of the six voxel selection methods can be found in the text. * represent significant difference exists between linear and RBF SVM classification results.

The classification results for linear and RBF SVM under six voxel selection schemes and two types of feature spaces (with PCA and without PCA) were shown in Figures 1 and 2. The accuracy was calculated as the ratio of the number of correctly classified scans for each of all categories to the total number of the scans of all categories. The classification accuracy was calculated for each subject, and the results averaged across subjects were presented in figures. The averaged time-consuming across subjects were shown in Tables 1 to [Table pone-0017191-t002] 3.

**Table 1:** The training and testing time for linear and RBF SVM across the 14 subjects when all the selected voxels were treated as features under different voxel selection methods (standard deviations were given in parentheses).

Voxel selection	Computational expense (in seconds)			
	Linear SVM		RBF SVM	
	train	test	train	test
RN*	5.88(2.20)	0.08(0.03)	148.02(52.99)	0.11(0.05)
**RF**	8.32(2.43)	0.11(0.04)	207.22(56.67)	0.15(0.04)
**RN**	12.07(1.84)	0.17(0.03)	297.05(40.39)	0.23(0.04)
**WF**	55.10(4.04)	0.99(0.46)	1284.47(707.91)	1.28(0.59)
WN*	80.69(51.26)	1.34(0.83)	1883.94(1179.89)	1.76(1.05)
**WN**	143.69(43.03)	2.35(0.72)	3271.09(931.11)	2.95(0.84)

**Table 2 pone-0017191-t002:** The training and testing time for linear and RBF SVM across the 14 subjects when principal components accumulatively accounting for 95% of the total variance of the original selected voxels were treated as features under different voxel selection methods (standard deviations were given in parentheses).

Voxel selection	Computational expense (in seconds)			
	Linear SVM		RBF SVM	
	train	test	train	test
RN*	0.82(0.13)	0.016(0.010)	20.55(2.24)	0.012(0.009)
**RF**	0.82(0.11)	0.010(0.008)	20.52(2.00)	0.014(0.007)
**RN**	0.97(0.10)	0.006(0.008)	23.20(1.95)	0.017(0.008)
**WF**	1.36(0.15)	0.015(0.010)	30.56(2.72)	0.020(0.007)
WN*	1.45(0.18)	0.019(0.009)	32.21(3.29)	0.022(0.008)
**WN**	1.60(0.10)	0.021(0.008)	35.19(1.77)	0.028(0.007)

**Table 3:** The averaged computational expense for linear and RBF SVM across the 14 subjects when the preprocessing time, the training and the testing time were reported together (standard deviations were given in parentheses).

Voxel selection	Computational expense (in seconds)			
	PCA space		Voxel space	
	Linear SVM	RBF SVM	Linear SVM	RBF SVM
RN*	11.88 (0.68)	32.09 (2.85)	26.08 (4.47)	164.13(52.09)
**RF**	18.57 (1.03)	32.46 (2.60)	45.35(6.38)	233.46(52.33)
**RN**	20.65 (0.76)	37.79 (2.52)	50.42(6.32)	338.63(32.70)
**WF**	28.91 (4.92)	57.56 (6.51)	79.91(35.90)	1243.60(631.21)
WN*	27.25 (8.64)	61.37 (10.64)	113.73(58.67)	1939.00(1193.85)
**WN**	41.09 (8.02)	73.15 (10.16)	183.06(48.84)	3308.36(936.12)

### Comparison of classification accuracy of different masks

#### Case 1: original selected voxels were used as input features for classifiers

The average number of voxels over the 14 subjects for each voxel selection scheme was shown in Figure 1 (A). The classification accuracy of the linear and RBF SVM classifier was shown in Figure 1(B). In this case, the original selected voxels for each mask were treated as features without any further feature selection or extraction. For linear SVM, the mean classification results across the 14 subjects were 66.52%, 66.48%, 66.82%, 70.87%, 63.69% and 67.56% for RN*, RF, RN, WF,WN*and WN masks respectively. Friedman's chi-square had a value of 13.037(df = 5, N = 14) and showed significant difference among the six voxel selection methods (p = 0.023). Clearly linear SVM achieved the best classification accuracy under the voxel selection scheme of WF (post-hoc test for the Friedman test [19], 0.05 level, Figure S1 (A)). Similarly, for RBF SVM, the mean classification results across the 14 subjects were 71.02%, 69.83%, 69.61%, 70.20%, 64.10% and 60.49% for the corresponding masks. Friedman's chi-square had a value of 21.463 (df = 5, N = 14) and also showed significant difference among the six voxel selection methods (p = 0.001). In this situation, RN*, RF, RN and WF voxel selection schemes achieved better results (post-hoc test, 0.05 level, Figure S1 (B)). All of the classification results were far above the chance level (25%).

#### Case 2: 95% of the PCs were kept as input features for classifiers

**Figure 3:**
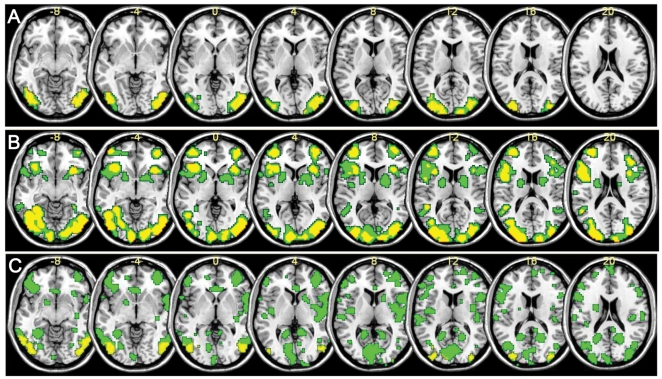
The masks for one representative subject (slices −8∼20). (A) RF mask and RN mask. Green: RN mask only; yellow: RF mask, which is also the overlap between RF and RN masks. (B) WF mask and WN mask. Green: WN mask only; yellow: WF mask, which is also the overlap between WF and WN masks. (C) RN* mask and WN* mask. Green: WN* mask only; yellow: RN* mask, which is also the overlap between RN* and WN* masks.

In this case, PCA was applied to reduce the dimensionality of the data and filter out noise before classification. We varied the number of PCs for each voxel selection schemes to reserve 95% of the variance of the original voxel space. The average number of PCs for each voxel selection scheme was shown in Figure 2(A). The classification results of the linear and RBF SVM classifier were shown in Figure 2(B). For linear SVM, the mean classification results across the 14 subjects were 64.14%, 62.54%, 63.02%, 67.67%, 64.40%, 67.11% for RN*, RF, RN,WF, WN* and WN masks (see Figure 3 for one representative subject of the six different brain masks) showing again significant difference among these masks (Friedman's chi-square 16.043,df = 5, N = 14, and p = 0.007). It's obvious that, with PCA, linear SVM under WF and WN voxel selection methods performed better than other brain masks (post-hoc test, 0.05 level, Figure S1(C)). Similarly, for RBF SVM, the mean classification results across the 14 subjects were 68.12%, 68.49%,67.86%,70.09%,66.22%, and 68.27% for the corresponding masks, no significant performance by this method among different masks (Friedman's chi-square 7.195,df = 5, N = 14, p = 0.207). All of the classification results under different masks were also far above the chance level (25%).

### Comparison of classification accuracy of linear and RBF SVM

#### Case 1: original selected voxels were used as input features for classifiers

The results indicated that RBF SVM performed better than linear SVM under three of the six voxel selection conditions as examined using Wilcoxon signed-rank test. In fact, the classification accuracy of RBF SVM was significantly better than linear SVM when using RN*, RF and RN masks (z = 3.114, p = 0.002; z = 2.921, p = 0.003; z = 2.973, p = 0.003 respectively). On the other hand, when using WN mask, linear SVM outperformed RBF SVM significantly (z = 3.235, p = 0.001) (Figure 1(B)). No significant difference existed between linear and RBF SVM under WF and WN* voxel selection schemes. In general, the classification accuracy of RBF SVM declined with the increasing of the number of voxels; it was superior to linear SVM in the relative low dimensional feature space (voxel space), and inferior to linear SVM in the relative high dimensional feature space.

#### Case 2: 95% of the PCs were kept as input features for classifiers

The average results showed that RBF SVM performed better than linear SVM under all the voxel selection conditions. Wilcoxon signed-rank test (one-tailed) was also performed on the classification accuracy, and RBF SVM became significantly better than linear SVM when used RN*, RF, RN, WF, and WN* masks (z = 3.015,p = 0.001; z = 3.016,p = 0.001; z = 3.007,p = 0.001; z = 1.923,p = 0.027; z = 1.890, p = 0.029 respectively), while for WN mask, RBF SVM outperformed linear SVM but not significantly(z = 1.354,p = 0.09) (Figure 2(B)).

### Computational expenses of different classifiers

Since we aimed to find the best combination of the voxel selection methods and the kernels of SVM classifiers, we compared the computational complexity measured with the amounts of time needed (training time and testing time respectively) for linear and RBF SVM classifiers here. Tables 1 and 2 listed the times in second without and with PCA respectively. The preprocessing time was not included here, e.g. the reading of the training and testing data, the standardizing of the features. In Table 3, the total computational cost (including the preprocessing time, the training time and the test time) was reported. The time reported was the average across 14 subjects (Windows XP, Intel Core 2 Duo CPU, 3.25G RAM, Matlab 7.0).

#### Case 1: original selected voxels were used as input features for classifiers

For both linear and RBF SVM, the training time was much longer than the testing time. RBF SVM was more time consuming than linear SVM under the same voxel selection schemes, the time cost difference mainly came from the training phase. We could see that the classification accuracy of linear SVM under WF mask was similar with RBF SVM under RN*, RF, RN and WF masks. Considering the overall time cost as shown in Table 3, linear SVM ran faster than its counterpart significantly for comparable classification results.

#### Case 2: 95% of the PCs were kept as input features for classifiers

Apparently, linear SVM was faster than RBF SVM under the same voxel selection method, and when PCA was used to reduce the dimensionality. It was very obvious and natural that the PCA dimension reduction shortened the time significantly compared to when all the voxels were used. In addition to the reduced computational time, the use of PCA was also associated with better performance of RBF SVM than linear SVM for 5 of the 6 masks (except WN) (Figure 2(B)).

## Discussion

The present study aimed to investigate the overall performance of linear and RBF SVM for fMRI classification together with voxel selection schemes on classification accuracy and the associated computational cost. Objective and explicitly results about the classification accuracy and the amounts of times needed for linear and RBF SVM under six voxel selection schemes and two types of feature spaces were given. In the following, we will discuss several aspects of our findings.

### Classification accuracy of linear SVM under different masks

Our study explicitly investigated the influence of voxel selection schemes for linear SVM on fMRI classification.

Firstly, linear SVM performed better when relative large number of voxels were included as features. As shown in Figure 1, linear SVM performed better on WF and WN masks than the other four brain masks. WF and WN masks are the two largest brain masks (except WN*) which select almost any useful voxel (voxels were selected through the whole brain). Our results also showcased the inability of the linear classifiers to identify the different objects from a small set of voxels (e.g. RF).

Secondly, the inclusion of voxels that were not maximally activated for one visual object when compared to others increased classification accuracy. Intuitively, we would assume the voxels that are more active for one category of object than the others may contain more information for classification. Our linear SVM results, on the contrary, showed that this kind of voxel selection methods (WN* and RN*) were not superior to the other methods in terms of accuracy of classification. This finding is similar with the study of Haxby et al., which reported that even the non-maximal responses carry category-related information, and thus be useful for classification [7].

Thirdly, FWE is a method that selects more relevant voxels for classification while the one without multiple comparison correction may include voxels with somewhat redundant information or noise. From Figure 3 we could see the spatial location and number of voxels varied significantly with and without correction. Voxels selected with FWE correction (RF, WF) were always a subset of those selected without correction (RN, WN). The classification accuracy under RF was not inferior to RN, indicative that additional voxels selected without correction provided no new information; the classification accuracy under WF was superior to WN, implicative that some of the voxels chosen without correction may actually provided no useful information for classification but rather contributed noise. As for the reason, we could see from the classification results of linear SVM with and without PCA (Figure1 (B), Figure2 (B)): when all the selected voxels were treated as features, the classification results under RF, RN and WF voxel selection schemes were significantly better than that when part of the PCs were kept as features (z = 2.794, p = 0.005; z = 2.605, p = 0.009; z = 2.417,p = 0.016 respectively by Wilcoxon signed-rank test), which was suggestive that the discarded PCs by PCA procedure may not be purely noise; while for WN voxel selection method, PCA did not weaken the classification results. One possible reason was that the unimportant features (noise, e.g.) were discarded while the informative ones kept.

In addition, all the voxel selection methods discussed above were designed to find out the active voxels during visual attention tasks. However, negative blood oxygenation level-dependent responses (deactivation) were also found in humans or animals under different tasks [20]–[23], which suggested that the deactivation voxels may also contribute to the classification of different cognitive tasks. The inclusion of deactivation voxels as features may enhance the decoding performance of classifiers, but no profound studies was conducted here.

### Classification accuracy of RBF SVM under different masks

Similar as linear SVM, two conclusions could be drawn: (1) Selection of voxels with FWE correction was shown to be adequate for classification. (2) Voxels that were not maximally active for one visual object stimulus in contrast to others were also useful for classification. By Wilcoxon signed-rank test, we found that RN* and WN* did not outperform RN and WN (z = 1.433, p = 0.152; z = 0.874, p = 0.382 respectively). The results also supported that those voxels that were not significantly more active for one category of object than others should have a contribution to the discrimination of brain states.

Different from linear SVM, the best classification results for RBF SVM were achieved when relative smaller voxels were used as features; with the number of voxels increased, the classification accuracy became worse (Figure 1). For RF, RN and WF, PCA procedure did not deteriorate the classification result significantly just as when we used linear SVM. It can be explained by the learning ability of RBF SVM, which is more powerful especially when the classification information was not sufficient. Besides, under WN voxel selection method, the classification result for RBF SVM became better after PCA because the features dimension became small.

### Classification accuracy comparison between Linear SVM and RBF SVM

We conducted the classification result comparison between the linear and non-linear RBF SVM combined with voxels selection when all the selected voxels were treated as features. Our findings (Figure 1) indicated that RBF kernel SVM outperformed linear SVM significantly in the relative low dimensional feature space (i.e. when the voxels were selected under the schemes of RN*, RF and RN), while linear SVM with enough input voxels (i.e. when the voxels were selected under the schemes of WN) got better classification accuracies than the RBF kernel SVM.

Logically, when the number of features is very large, there is a high likelihood that the data are linearly separable in the original space, and therefore no need to map the data in to a higher dimensional space [24]. On the other hand, non-linear SVM provides the possibility to map the linearly non-separable data in a low dimensional space (low number of voxels) into a space of very high dimension for better linear separability. Norman et al. [5] pointed out that the key difference between non-linear and linear classifiers was that non-linear classifiers could respond to high-level feature conjunctions in a way that differed from their response to individual features. That explains the better performance of RBF SVM in the relative lower dimensional space.

The Vapnik-Chervonenkis dimension (VC-dimension) [25] measures the capacity of classification for SVM algorithms. It is an important tool to understand the capacity of different kernels of SVM under different circumstances and is defined as the cardinality of the largest set of points that the machine learning algorithm can shatter. The VC-dimension for linear SVM in m-dimensions feature space is m+1, for RBF kernel SVM is infinity. Apparently, the VC-dimension for linear SVM increased with the number of voxels when all the selected voxels were treated as features. Under WF, WN* and WN voxel selection methods, the learning capacity of linear SVM was possibly good enough, and comparable to the RBF SVM, that is why linear SVM performed equal or even better than RBF SVM with the increased number of the voxels. In addition, the ratio of support vectors to training vectors for RBF SVM under WF, WN* and WN were 92.30%, 95.13% and 99.26% respectively (averaged across all the subjects), suggesting that RBF SVM may suffer from overfitting with the increase of the number of voxels.

Multidimensional scaling (MDS) [26] is an algorithm for dimensionality reduction. It preserves the original distances in the original high dimensional space. For a better explanation of our results, we used MDS to map the data to a 2-dimensional space for visualization purpose. As the present study was a four-class classification problem, we employed Pair-Wise approach to compute separation space that discriminated every pair of classes (according to LIBSVM, http://www.csie.ntu.edu.tw/~cjlin/libsvm). Hence 4(4-1)/2 = 6 binary classifiers were required. So the distributions of the training examples, the support vectors, and the decision surface of the linear classifiers were shown for the six two-class classifiers respectively under RN*, RF, RN, WF, WN* and WN masks (Figure S2, S3, S4, S5, S6 and S7) (MDS was accomplished by using the Matlab Toolbox for Dimensionality Reduction. http://homepage.tudelft.nl/19j49/Matlab_Toolbox_for_Dimensionality_Reduction.html, and the visualization work was accomplished using the plot function written by Steve Gunn, http://www.isis.ecs.soton.ac.uk/resources/svminfo/). Corresponding results of RBF SVM classifiers were shown for the six two-class classifiers respectively under RF mask (Figure S8). The results for linear SVM under RN*, RF and RN are similar. From Figure S2, S3 and S4, we can see the brain states when subjects viewed (A) house and face, (C) house and cat, (D) face and car are approximately linear separable, when subjects viewed (B) house and car, (E) face and cat, (F) car and cat are linear non-separable. It's also interesting to notice (B) in Figure S8, the non-linear classifier performed well in discriminating the house and car which was shown to be linear non-separable in Figure S2. The linear separability for some kind of cognitive tasks and linear non-separablity for others in the relative low dimensional space may explain the classification results shown in Figure 1. In the relative lower dimensional space, RBF SVM outperformed linear SVM; this is because linear non-separable cognitive states existed. In the relative higher dimensional space (WF and WN), the brain states when subjects viewed face and cat (Figure S5 (E), S7 (E)) became approximately linear separable too, and the classification capacity for linear SVM became strong while nonlinear SVM was unnecessary. This could be reasons that the linear SVM and RBF SVM performed almost equivalently under WF mask, and linear SVM even outperformed its nonlinear counterpart in a higher dimensional space (WN). From Figure S6 we could see when using WN* voxel selection method, all the six two-class cognitive brain states became linear non-separable, which may explain why the classification results under WN* mask were the worst among the six voxel selection methods (Figure 1). In short, voxel selection did have an important impact on the classification problem.

In the context of fMRI data classification, several studies have compared the performance of linear and nonlinear SVM (with different kernels) [14] [18] [27]. Although many conceivable sources of non-linearity exist in neural signals, Cox and Savoy's study illustrated that non-linear SVM (polynomial kernel) did not show a clear benefit versus linear SVM [14]. However, in concert with the present result, Cox et al. found that in the relatively lower dimensional feature space, non-linear SVM outperformed linear SVM; with the increasing of the features, linear SVM achieved better result suggesting that non-linear SVM possibly suffered from overfitting. Linear and RBF SVM have been directly compared in decoding the category of visual objects (three groups of two-class classification problem) from response patterns in human early visual cortex and inferior temporal cortex [18]. In the relatively high dimensional feature space, RBF SVM performed significantly worse than linear SVM. However, in the low dimensional feature space, a significant difference was not found between these two classifiers. That was possible since many differences existed between their study and ours, e.g. the types of visual stimuli, the ROIs, the feature selection rule, the number of subjects, and the criterion for assessing significant differences, which would influence the performances and the comparison results of the classifiers.

### Computational expense comparison between linear and RBF SVM

Besides classification accuracy, the amounts of times needed for classifier construction and for classification are also an important factor for consideration. In this regard, a computational expenses were compared between linear and RBF SVM (Table 1, 2 and 3). Results showed that for both linear and RBF SVM, the training time was much longer than the testing time. Regardless if the feature space consisted of voxels or PCs, under the same voxel selection method, linear SVM was significantly faster than non-linear SVM. Overall, the time cost difference mainly came from the training phase as the testing time for both linear and RBF SVM was short and practically feasible. For researchers who are interested primarily the real-time fMRI classifications (assuming the classifier has already been trained, e.g.) their decision should be based on the classification accuracy.

Time reported in Tables 1 and 2 did not include the image preprocessing time as they are the same for any method. If the preprocessing time was also considered (e.g. the reading of the training and testing data, the standardizing of the data, the PCA if necessary) (Table 3), we could see linear SVM under WF voxel selection method was a better choice when all the selected voxels were treated as features. When the features were the PCs of the original voxels, linear SVM could achieve almost the same classification accuracy with RBF SVM only when the mask (WN) contained the largest number of voxels among the six voxel selection method. Considering the classification accuracy and the computation time holistically (Table 3), RBF SVM classifier with RN* or RF mask was a better choice for brain states classification. On the other hand, although the classification result of linear SVM using the WF mask was inferior to classification of RBF SVM with the same mask, the results were still acceptable and may be preferred in application due to their computationally less expensive properties.

Beyond all the discussions above, one more thing should be mentioned: as one of the commonly used preprocessing step for fMRI data, space smoothing may destroy useful information for classification. It has been shown that linear SVM was less sensitive to smoothing compared with FLD and Canonical Variates Analysis [17] [27]. What the impact of smoothing for linear and RBF SVM when using PCA was also an interesting question which we may investigate in the future.

To summarize, the present work provides the first empirical result of linear and RBF SVM in classification of fMRI data, combined with a variety of voxel selection schemes. Both linear and RBF SVM can achieve good classification accuracy under appropriate voxel selection method. RBF SVM performed better than linear SVM in a relative lower dimensional space, while linear SVM outperformed RBF SVM in a relative higher dimensional space. Taking both the classification accuracy and the amounts of time needed into consideration, linear SVM with relative more voxels as features and RBF SVM with small set of voxels (after PCA) could achieve the better accuracy with reasonable computational expenses. These objective results may be informative for researchers searching for desired classification accuracy or computation expense.

## Materials and Methods

### Ethics Statement

The study was approved by the Institutional Review Board of Beijing Normal University (BNU) Imaging Center for Brain Research, National Key Laboratory of Cognitive Neuroscience and Learning. All subjects gave written informed consent.

### Subjects and fMRI data acquisition

Volunteers were recruited from BNU, Beijing, China. 14 healthy college participants were included in the study (6 males and 8 female s).

A 3-T Siemens scanner equipped for echo planar imaging (EPI) at the Brain Imaging Center of BNU was used for the image acquisition. For each participant, functional images were collected with the following parameters: repeat time (TR) = 2000 ms; echo time (TE) = 30 ms; 32 slices; matrix size = 64×64; acquisition voxel size =  3.125×3.125×3.84 mm; flip angle (FA) = 90°; field of view (FOV) = 190∼200 cm. In addition, a high-resolution, three-dimensional T1-weighted structural image was acquired (TR = 2530 ms; TE = 3.39 ms; 128 slices; FA = 7°; matrix size = 256×256; resolution = 1×1×1.33 mm).

### Stimuli and experimental procedure

**Figure 4:**
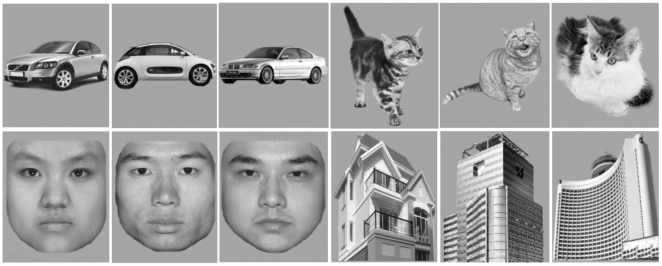
Examples of stimuli. Subjects had to press a button with their left or right thumb as long as images were repeated consecutively.

The experiment was designed in a blocked fashion. All subjects participated in 8 runs and each run consisted of 9 blocks, with 4 task blocks and 5 control blocks. Subjects viewed objects from four categories (houses, faces, cars or cats) (Figure 4). During each task block which lasted for 24 s, 12 stimuli belonging to one particular category were presented, and subjects had to press a button with their left or right thumb if any image repeated itself consecutively to ensure that participants were paying attention while they viewed the images [7]. Two identical images were displayed consecutively 2 times randomly during each block. Each stimulus was presented for 500 ms followed by a 1500 ms blank screen. Control blocks were 12-s fixation in the beginning of a run and at the end of every task block. Each kind of objects were presented only one time during each run, and the order of them were counterbalanced in the whole session which lasted 20.8 minutes. Thus, 384 images were acquired for the image attention tasks, 96 for each category.

The stimuli were gray-scale images for four categories of objects with the same size. During each task block, the 12 pictures presented were randomly chosen from 40 pictures of one particular category. Although the same picture sets of objects were used for both training and testing, the chance for the two sets of 12 pictures to be identical was almost impossible (Probability is 5.9605×10^−20^).

### Data preprocessing

We used SPM2 (http://www.fil.ion.ucl.ac.uk/spm/) to process the imaging data. It mainly contains 3 steps: realignment, normalization and smoothing. Subjects were preprocessed separately. In the beginning, the first 3 volumes were discarded as the initial images of each session showed some artifacts related to signal stabilization (according to the SPM2 manual). Images were realigned to the first image of the scan run and were normalized to the Montreal Neurological Institute (MNI) template. The voxel size of the normalized images was set to be 3*3*4 mm. At last, images were smoothed with 8 mm full-width at half maximum (FWHM) Gaussian kernel. The baseline and the low frequency components were removed by applying a regression model for each voxel [17]. The cut-off period chosen was 72 s.

### Voxel selection schemes

Voxels were selected within the whole brain or ROIs defined by using the WFU Pickatlas (http://www.fmri.wfubmc.edu). Previous studies have shown selective activation for different kinds of objects in the visual cortex [14], [28], [29]. Four ROIs were chosen here: fusiform gyrus, inferior temporal gyrus, inferior occipital gyrus and middle occipital gyrus. Two methods were used to determine the thresholds when multiple comparisons were carried out throughout the whole brain as well as within ROIs: one way the family-wise error (FWE) correction to control the probability of false rejection of un-active voxels among all hypotheses tested; the other way was to set the threshold without the multiple comparison correction. Both of them were implemented in SPM2. The P value was set to be 0.05 for FWE and 0.001 for no correction method. All the voxels above the thresholds were defined as active.

Voxels were selected in the following way for each subject separately (this procedure was equal to the process of producing a brain mask):

1. Whole brain and no correction (WN)
  Any voxels activated for each of the 4 categories of stimuli in the whole brain were selected, and the threshold was set without correction. All the chosen voxels were set to be 1, while the rest were set to be 0; thus, four masks were produced, one for each of the four object categories separately. A new mask was created that contained all the voxels activated for at least one category of objects (logic OR).
2. Whole brain and no correction* (WN*)
  Similar to the WN method, voxels were also selected through the whole brain and the threshold was set without correction. However, considering voxels activated stronger for one category than others may be more useful for classification; voxels that were activated stronger for one specific object category (such as houses) than the other three objects (such as faces, cars and cats) were selected in the whole brain without correction. A brain mask was produced, with all the selected voxels set to be 1 and the rest 0. Again, four masks were produced, one for each of the four object categories separately. And the logic OR mask was formed.
3. Whole brain and FWE correction(WF)
  Any voxels activated for at least one of the categories of stimuli exceeding the FWE corrected thresholds in the whole brain were selected.
4. ROIs and no correction (RN)
  Any voxels activated for one kind of stimuli (e.g. house) within all the ROIs above the threshold without correction were selected.
5. ROIs and FWE correction (RF)
  Any voxels activated for at least one of the categories of stimuli within all the ROIs were selected (FWE correction).
6. ROIs and no correction* (RN*)
  Like the WN* method described above, voxels that activated for one specific object category (such as houses) stronger than the other three objects (such as faces, cars and cats) were selected in all the ROIs without correction. FWE correction was too strict for selecting voxels for one category of stimuli activated stronger than the other three, so it wasn't used to set the threshold in the WN* and RN* voxel selection methods.

Overall, six different brain masks (0/1 mask) were produced for each single subject. Element by element multiplication operation was done between the preprocessed images of a single subject and each of the six brain masks. These fMRI series were then re-organized in to a new matrix (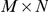), where M was the number of scans and N was the number of selected voxels (the voxels in a 3D volume image was re-arranged to a row vector). Here, selected voxels were treated as features, and volumes were samples. Each feature (a column in the M by N matrix) was standardized to have mean 0 and standard deviation 1.

As we were only interested in the task data in this study, we divided the fMRI data into two sets: the first 4 runs as the training data, and the last 4 runs as the test data. Thus, we had 192 samples (time series) for training and test respectively for each subject, 48 for each category. Note that the voxel selection schemes were applied to the training data to decide which voxels will be included for the training and for the testing datasets (i.e., voxel selection was not performed for the testing dataset independently).

Besides, as a commonly used dimensionality reduction approach, the impact of PCA [6], [17] was also investigated in this study. In some situation, the dimensionality reduction is very important, since when the number of input features is large, the computational expense will increase, especially for non-linear classifiers (Table 1). PCA procedure was conducted over the voxels in each of the 6 masks and PCs accumulatively accounting for 95% of the total variance of the original data were kept for the subsequent classification (Figure 2(A)). Again, like the voxel selection procedure, the PCA was estimated based on the training data and applied to the testing data. In other words, the test data was directly projected to the direction of the PCs.

### Support vector machine

Linear SVM is one method used in statistics and machine learning to find a linear combination of features which characterize or separate two or more classes of objects or events. Since the fMRI brain activity patterns associated with the object recognition may not be linearly separable [14], we also considered non-linear SVM. Non-linear SVM applies the kernel trick to maximum-margin hyperplanes; it classifies the fMRI feature mapped to the high-dimensional feature space where the feature may be non-linear in the original input fMRI data space become linearly separable. The adequacy of SVM relies on the proper selection of kernels, the one with the best classification accuracy is the classifier whose kernel function captures the distribution pattern of fMRI data.

SVM [25] has been used for the classification of brain states in a number of previous fMRI studies [14]–[18], [27]. Cox and Savoy [14] used linear kernel and polynomial kernel SVM to classify multiclass patterns of brain activation, and no significantly better performance was found for non-linear SVM. Here, we used another basic non-linear kernel function, radial basis function (RBF) kernel: 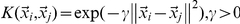. Suppose we have a two-class training set: 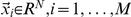, and the corresponding label set is 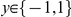, the purpose of SVM is to find the optimized solution to the following problem(in the mapped/projected space):



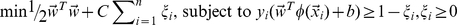



Here, 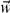 is a weight vector and b is an offset. The hidden non-linear function 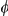 maps the training data into a higher dimensional feature space where the optimized hyperplane is calculated. Although nonlinear transformation is essential in SVM, we do not need to know this mapping explicitly, because only the dot product of feature vectors is used, i.e. 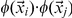, in both the training and test. A kernel function is defined as a function that corresponds to a dot production of two feature vectors in some expanded feature space. The nonlinear projection is contained in the kernel function: 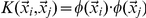, then in the higher dimensional feature space the inner production is accomplished by the calculations in the original space. 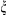 is the slack variable introduced for linearly un-separable training data which represents the distance for the misclassified training data to the margin boundary. C is the penalty parameter which makes a compromise between the number of misclassified samples and the complexity of the algorithm. For SVM, the kernel and the parameter C control model complexity. There are three reasons why RBF is a better choice [24]: First, RBF can handle the nonlinear relation between class labels and attributes. In addition, the linear kernel is a special case of RBF and the sigmoid kernel behaves like RBF for certain parameters. Secondly, the polynomial kernel has more hyper-parameters than the RBF kernel while the number of hyper-parameters can influences the complexity of model selection. Finally, compared with the polynomial kernel as well as the sigmoid kernel, the RBF kernel has less numerical difficulties.

Multi-class libsvm [30] was used to perform the classifications, in which 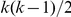 (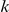 is the number of classes) two-class classifiers were trained, each of them contributed to the final decision by a simple voting mechanism. The procedure of classification is as follows [24]:

- Scale the attributes of training data to the range [-1, 1] linearly; then scale the attributes of the test data using the same scaling function of the training data. For example, suppose one attribute of training data was scaled from [-10, 10] to [−1, 1], the same attribute of the test data was scaled from [−9, 11] to [−0.9,1.1].
- Consider the RBF kernel 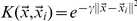

- Use 5 fold cross-validation to find the best parameter C and 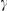. The range of C and 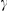 was 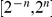, n = 1,2,…10.
- With the values of the parameters C and 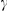 determined, whole training set was used to construct the SVM (i.e. to estimate the weight vector w), then a model was created for the test data.
- Evaluate the constructed SVM in term of classification accuracy based on the testing dataset.

## Supporting Information

Figure S1 **Comparison of each voxel selection methods against each other under the situations of (A) Linear SVM without PCA. (B) RBF SVM without PCA. (C)Linear SVM with PCA.** In each plot, entry (a,b) is positive (red) if the classification accuracy under voxel selection method a is larger than that of voxel selection method b significantly under the post-hoc test (0.05 level), and negative (blue) if the reverse is true. The critical values were 1.32, 1.21 and 1.28 for the three conditions respectively. The code for post-hoc analysis of Friedman test was provided by http://timo.gnambs.at/en/scripts/friedmanposthoc.
(TIF)

Figure S2 The distribution of the training examples and the support vectors (marked in white circle) when using linear SVM under RN* mask. (A) House vs. face, (B) House vs. Car, (C) House vs. Cat, (D) Face vs. Car, (E) Face vs. Cat, (F) Car vs. Cat.
(TIF)

Figure S3 **The distribution of the training examples and the support vectors (marked in white circle) when using linear SVM under RF mask.** (A) House vs. face, (B) House vs. Car, (C) House vs. Cat, (D) Face vs. Car, (E) Face vs. Cat, (F) Car vs. Cat.
(TIF)

Figure S4 **The distribution of the training examples and the support vectors (marked in white circle) when using linear SVM under RN mask.** (A) House vs. face, (B) House vs. Car, (C) House vs. Cat, (D) Face vs. Car, (E) Face vs. Cat, (F) Car vs. Cat.
(TIF)

Figure S5 **The distribution of the training examples and the support vectors (marked in white circle) when using linear SVM under WF mask.** (A) House vs. face, (B) House vs. Car, (C) House vs. Cat, (D) Face vs. Car, (E) Face vs. Cat, (F) Car vs. Cat.
(TIF)

Figure S6 The distribution of the training examples and the support vectors (marked in white circle) when using linear SVM under WN* mask. (A) House vs. face, (B) House vs. Car, (C) House vs. Cat, (D) Face vs. Car, (E) Face vs. Cat, (F) Car vs. Cat.
(TIF)

Figure S7 **The distribution of the training examples and the support vectors (marked in white circle) when using linear SVM under WN mask.** (A) House vs. face, (B) House vs. Car, (C) House vs. Cat, (D) Face vs. Car, (E) Face vs. Cat, (F) Car vs. Cat.
(TIF)

Figure S8 **The distribution of the training examples and the support vectors (marked in white circle) when using RBF SVM under RF mask.** (A) House vs. face, (B) House vs. Car, (C) House vs. Cat, (D) Face vs. Car, (E) Face vs. Cat, (F) Car vs. Cat.
(TIF)
