# Supplementary figures and images for: Comparative Study of SVM Methods Combined with Voxel Selection for Object Category Classification on fMRI Data
Source: PLoS One. 2011 Feb 16;6(2):e17191. doi: 10.1371/journal.pone.0017191 (PMC3040226; doi:10.1371/journal.pone.0017191)

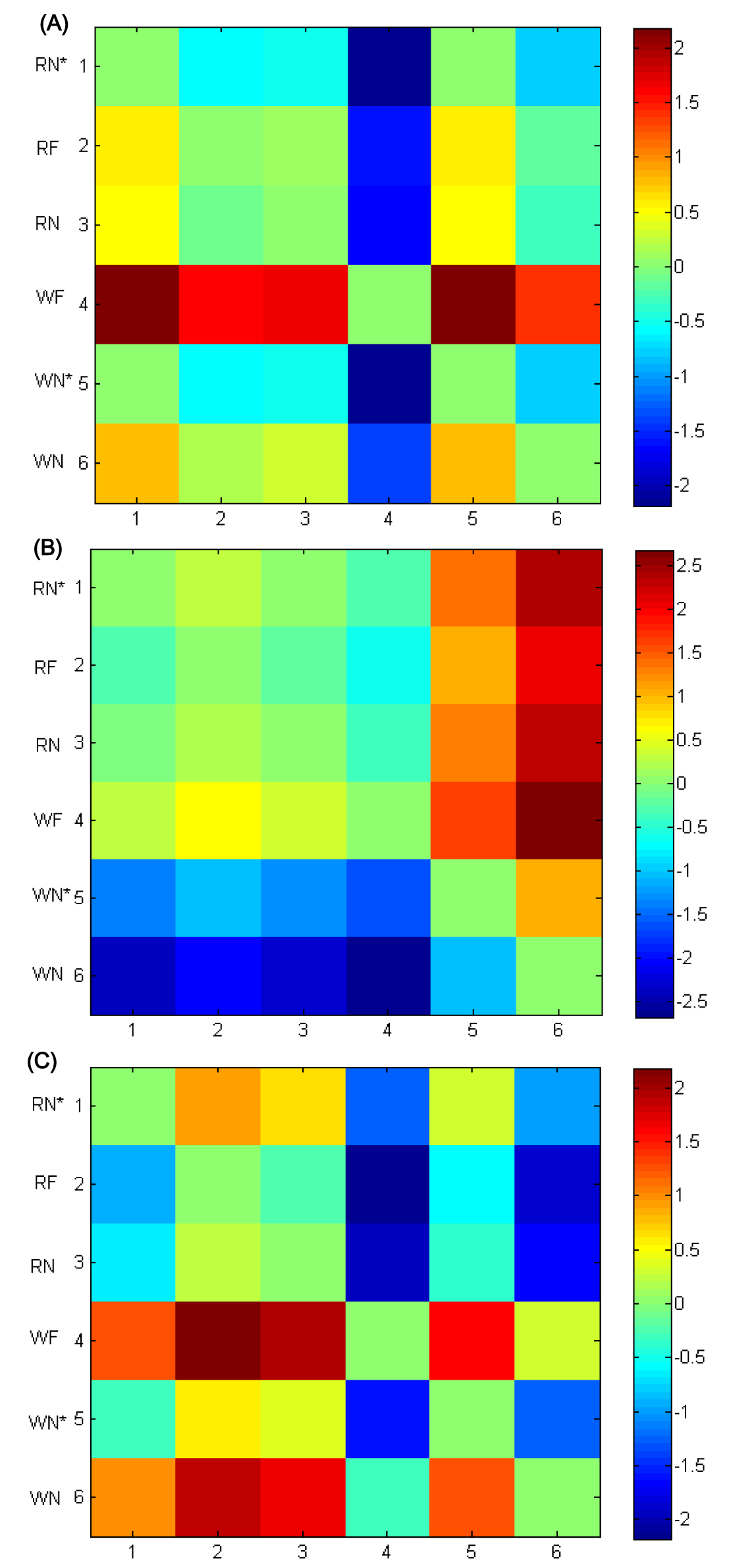

Supplement: Figure S1 — Comparison of each voxel selection methods against each other under the situations of (A) Linear SVM without PCA. (B) RBF SVM without PCA. (C)Linear SVM with PCA. In each plot, entry (a,b) is positive (red) if the classification accuracy under voxel selection method a is larger than that of voxel selection method b significantly under the post-hoc test (0.05 level), and negative (blue) if the reverse is true. The critical values were 1.32, 1.21 and 1.28 for the three conditions respectively. The code for post-hoc analysis of Friedman test was provided by http://timo.gnambs.at/en/scripts/friedmanposthoc. (TIF) [file pone.0017191.s001.tif]

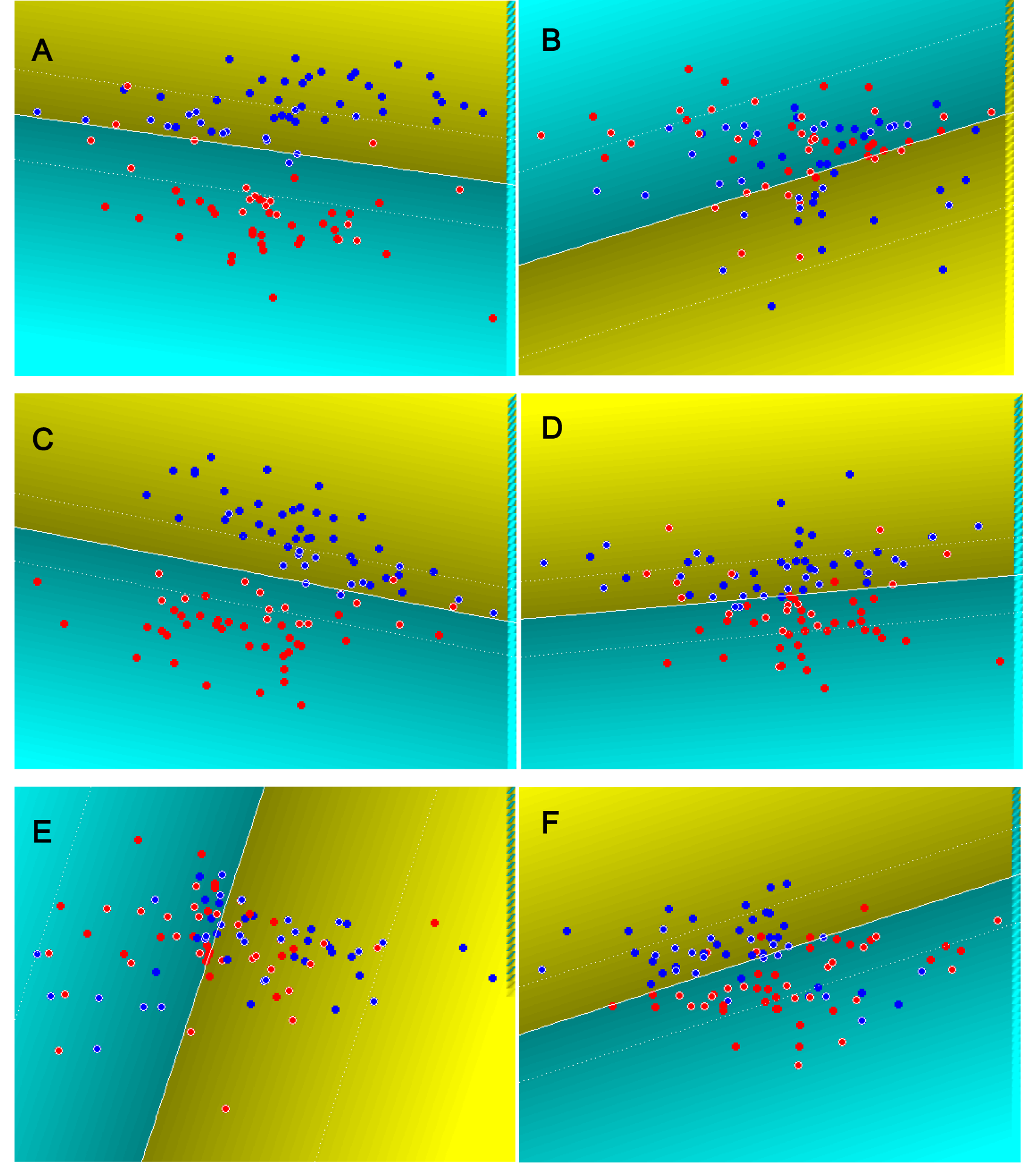

Supplement: Figure S2 — The distribution of the training examples and the support vectors (marked in white circle) when using linear SVM under RN* mask. (A) House vs. face, (B) House vs. Car, (C) House vs. Cat, (D) Face vs. Car, (E) Face vs. Cat, (F) Car vs. Cat. (TIF) [file pone.0017191.s002.tif]

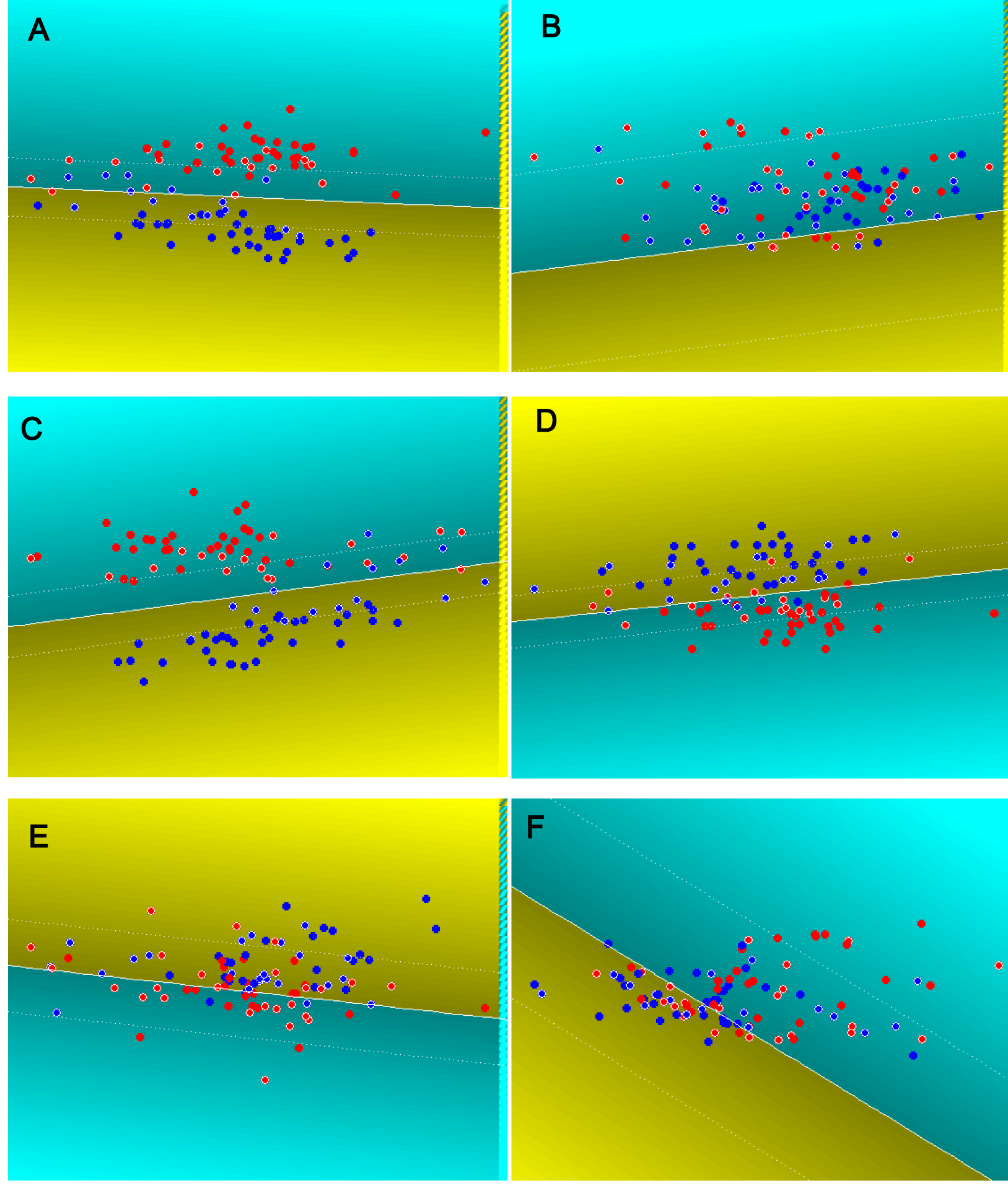

Supplement: Figure S3 — The distribution of the training examples and the support vectors (marked in white circle) when using linear SVM under RF mask. (A) House vs. face, (B) House vs. Car, (C) House vs. Cat, (D) Face vs. Car, (E) Face vs. Cat, (F) Car vs. Cat. (TIF) [file pone.0017191.s003.tif]

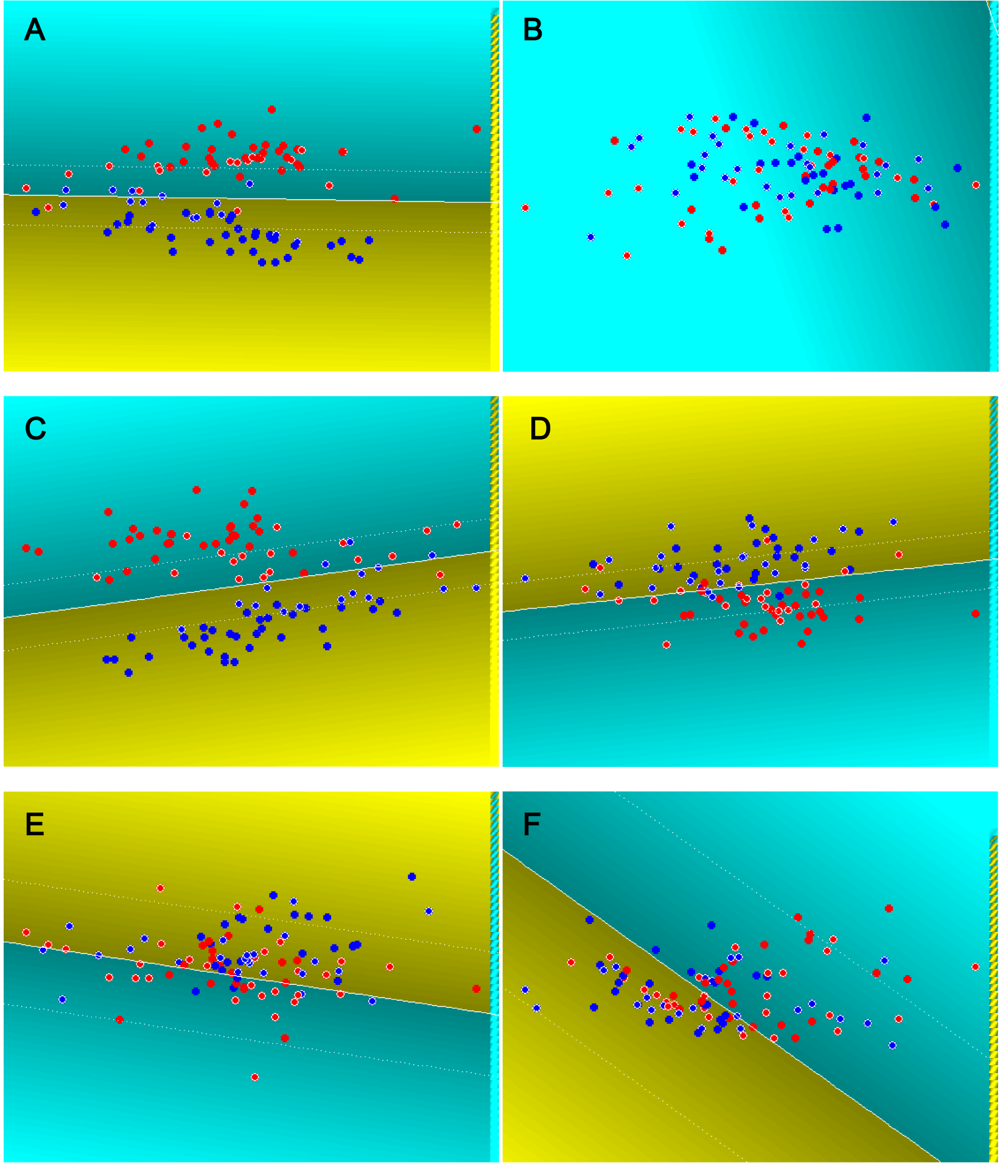

Supplement: Figure S4 — The distribution of the training examples and the support vectors (marked in white circle) when using linear SVM under RN mask. (A) House vs. face, (B) House vs. Car, (C) House vs. Cat, (D) Face vs. Car, (E) Face vs. Cat, (F) Car vs. Cat. (TIF) [file pone.0017191.s004.tif]

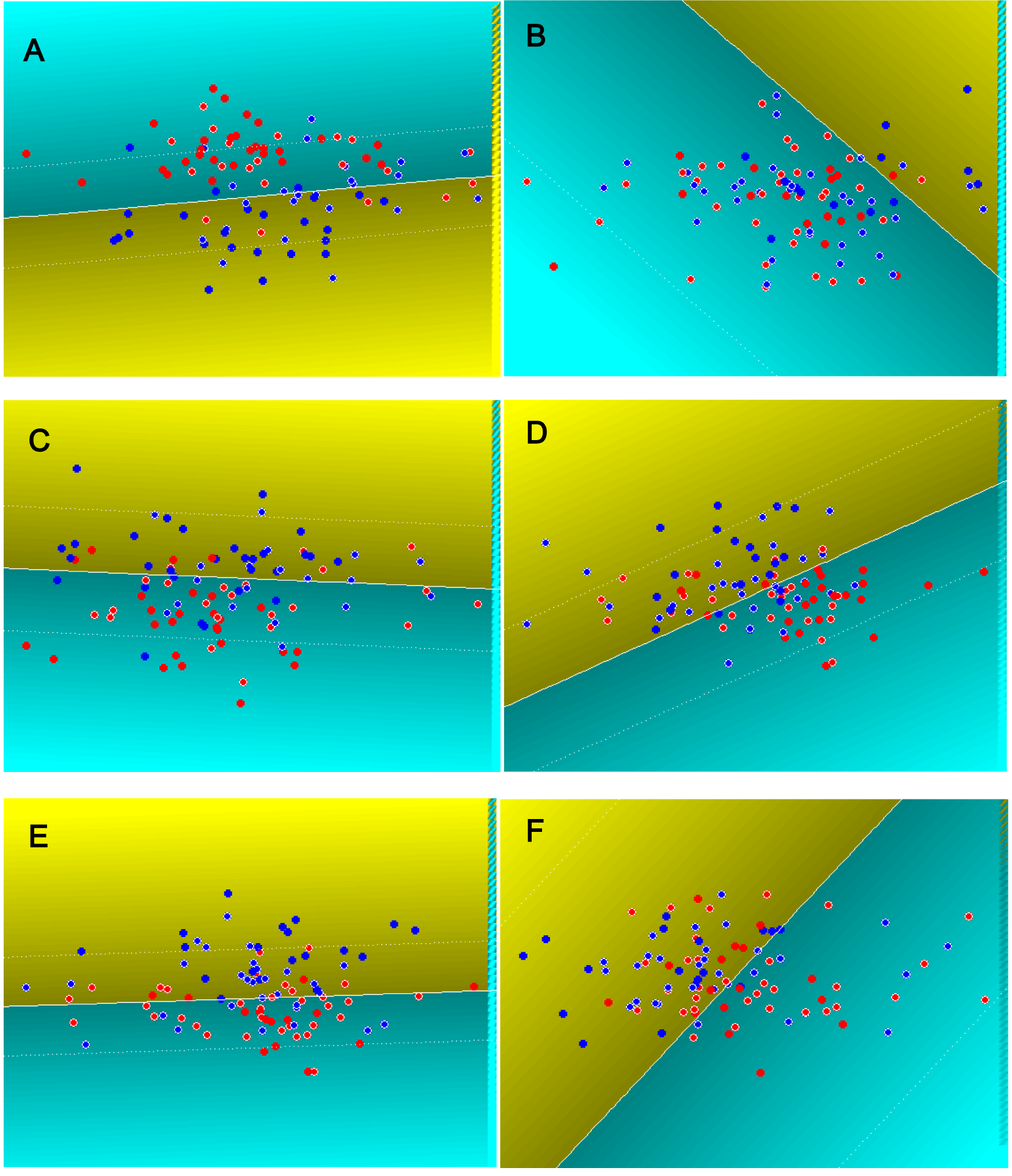

Supplement: Figure S5 — The distribution of the training examples and the support vectors (marked in white circle) when using linear SVM under WF mask. (A) House vs. face, (B) House vs. Car, (C) House vs. Cat, (D) Face vs. Car, (E) Face vs. Cat, (F) Car vs. Cat. (TIF) [file pone.0017191.s005.tif]

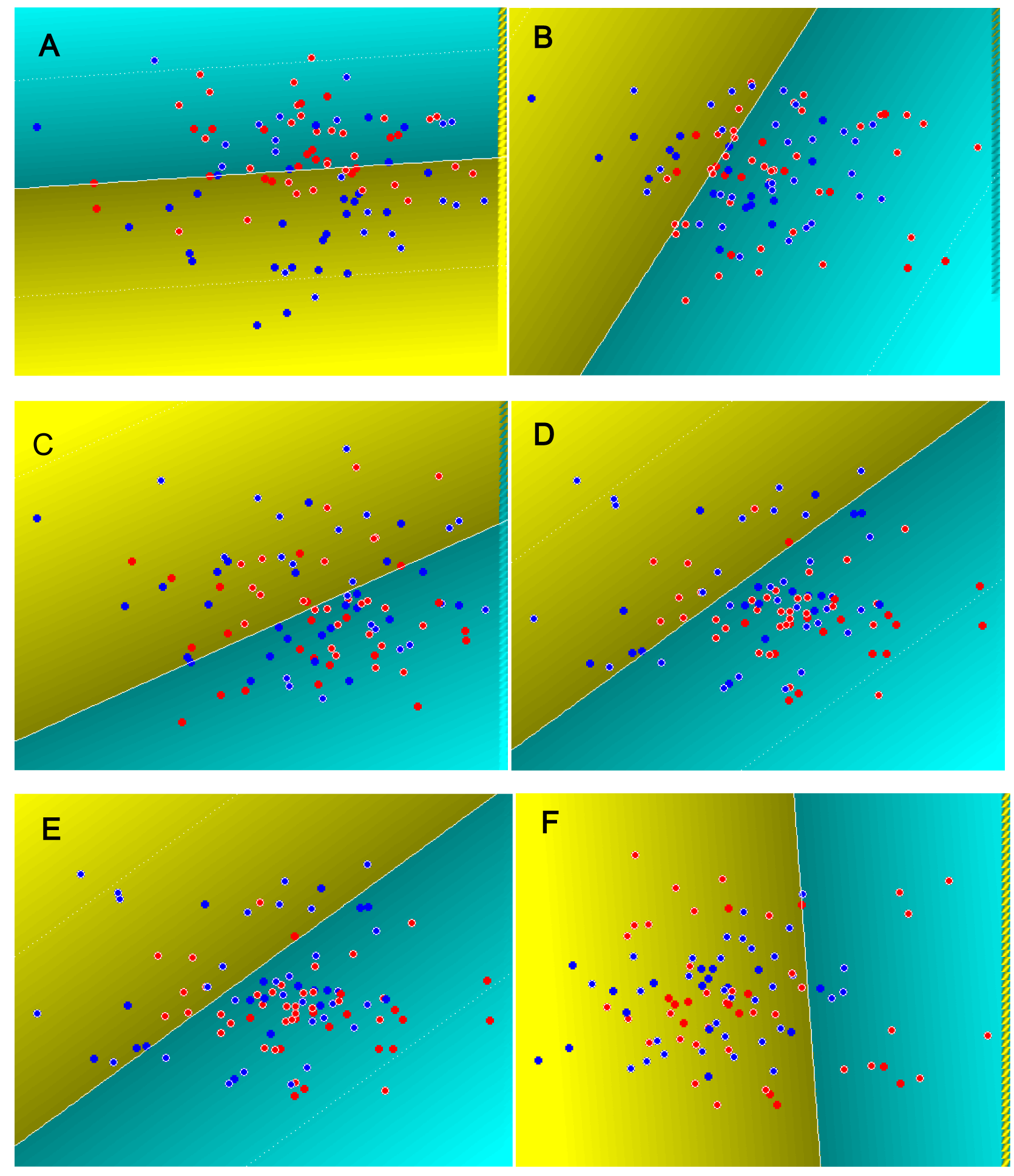

Supplement: Figure S6 — The distribution of the training examples and the support vectors (marked in white circle) when using linear SVM under WN* mask. (A) House vs. face, (B) House vs. Car, (C) House vs. Cat, (D) Face vs. Car, (E) Face vs. Cat, (F) Car vs. Cat. (TIF) [file pone.0017191.s006.tif]

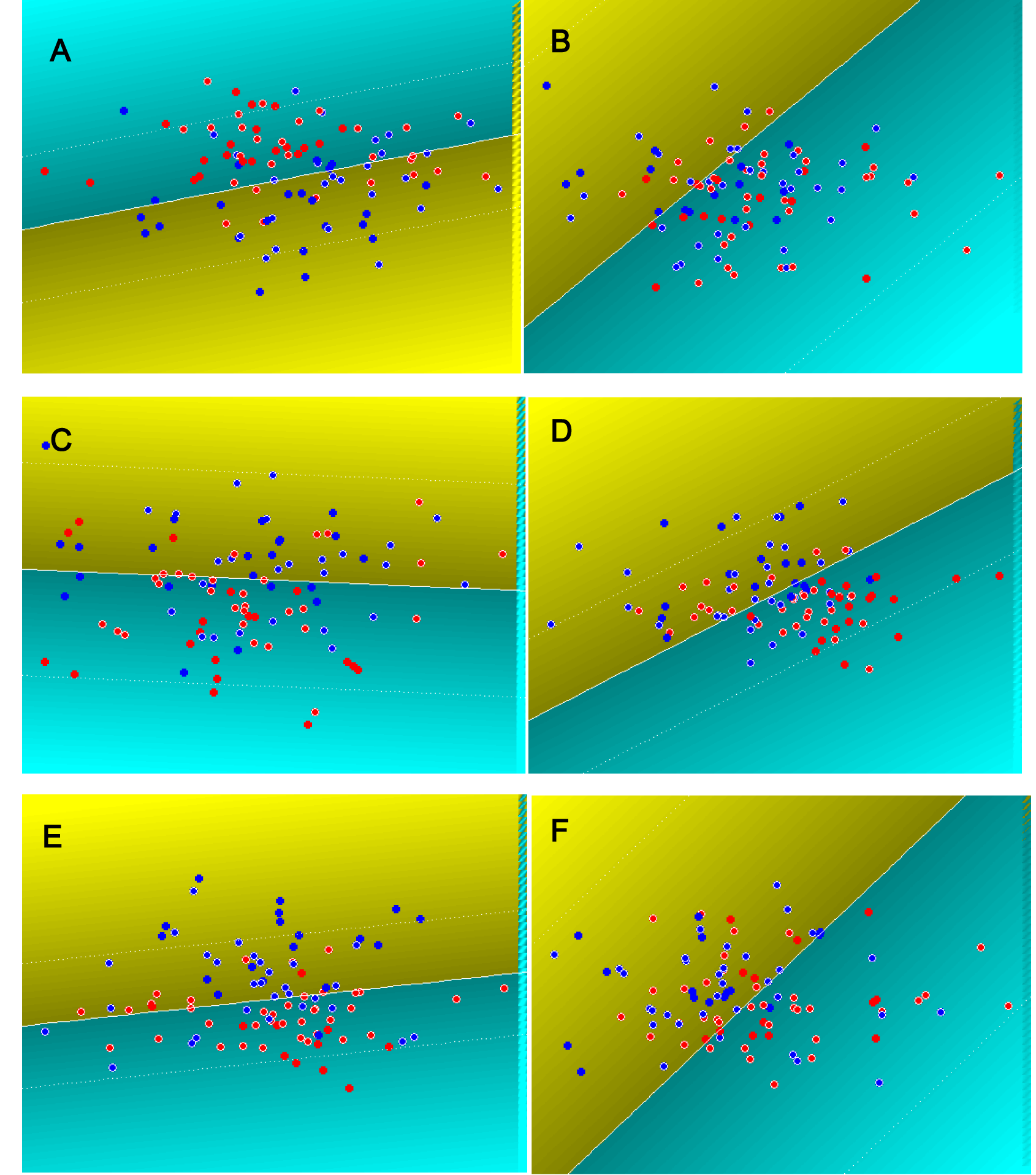

Supplement: Figure S7 — The distribution of the training examples and the support vectors (marked in white circle) when using linear SVM under WN mask. (A) House vs. face, (B) House vs. Car, (C) House vs. Cat, (D) Face vs. Car, (E) Face vs. Cat, (F) Car vs. Cat. (TIF) [file pone.0017191.s007.tif]

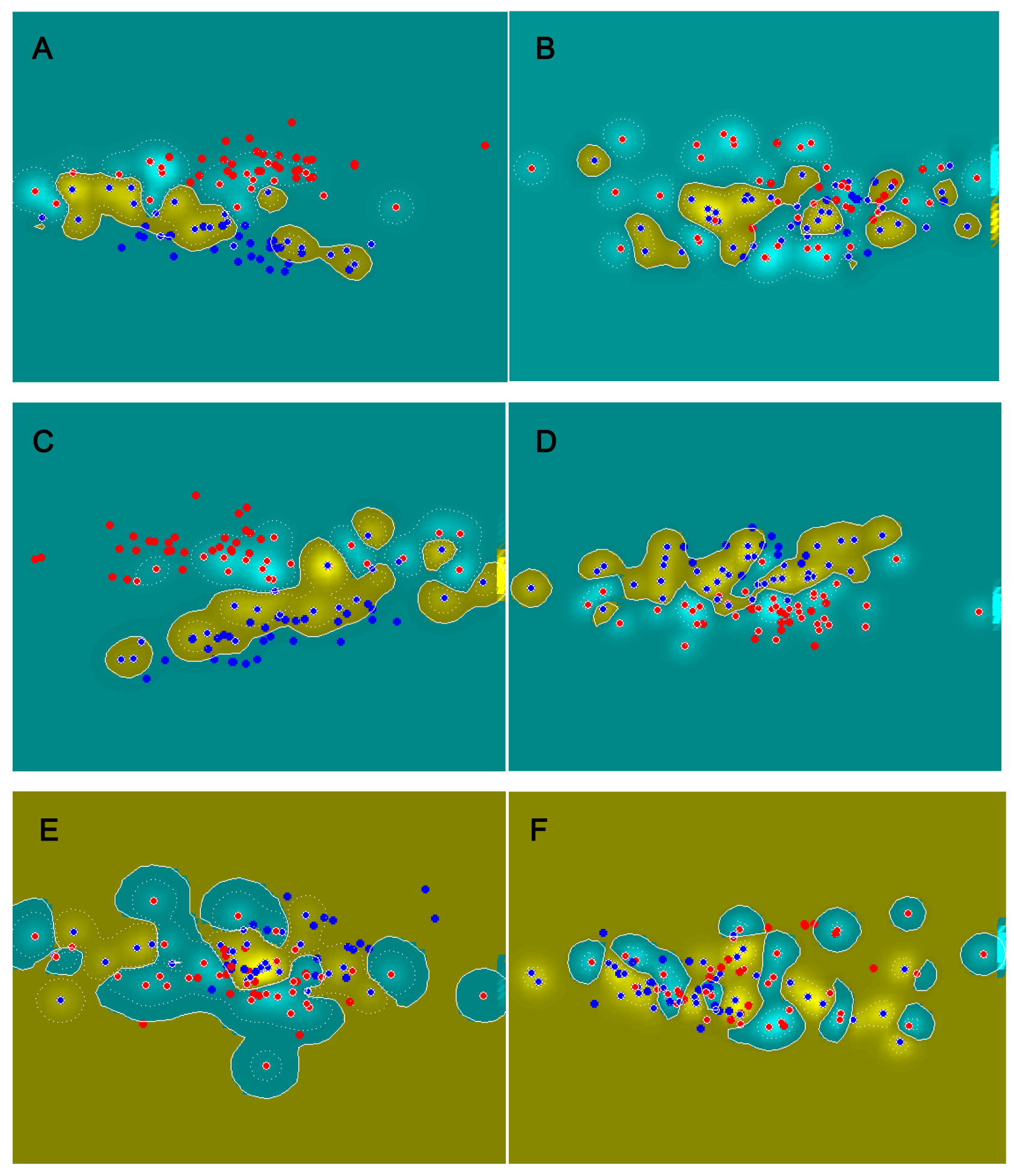

Supplement: Figure S8 — The distribution of the training examples and the support vectors (marked in white circle) when using RBF SVM under RF mask. (A) House vs. face, (B) House vs. Car, (C) House vs. Cat, (D) Face vs. Car, (E) Face vs. Cat, (F) Car vs. Cat. (TIF) [file pone.0017191.s008.tif]
